# Sum or mean in calculation of qualitative scoring methods using the Dragonfly Biotic Index, and an alternative approach facilitating conservation prioritization

**DOI:** 10.1038/s41598-024-62017-y

**Published:** 2024-05-18

**Authors:** Hana Šigutová, Petr Pyszko, Eva Bílková, Veronika Prieložná, Aleš Dolný

**Affiliations:** 1 Department of Zoology, Faculty of Science, Palacký University, Olomouc, Czech Republic; 2 Department of Biology and Ecology, Faculty of Science, University of Ostrava, Chittussiho 10, 710 00 Ostrava, Czech Republic

**Keywords:** Conservation biology, Freshwater ecology

## Abstract

Qualitative scoring methods are tools for rapid freshwater health assessments. Total score is often calculated as the sum or mean of the values of the species involved, with minor nuances in interpretation, but with significant implications. We used the Dragonfly Biotic Index (DBI) calculated on Central European odonate species to demonstrate these implications. Each species within a community has a score ranging from 0 (widespread generalists) to 9 (sensitive specialists). A total score is calculated as the sum of the scores of all species (DBI_sum_) or is calculated by dividing by species richness (DBI_mean_). Despite this duality, there has been little debate on either approach. Using simulated scenarios (high vs low richness, presence or absence of high- or low-scoring species), we tested the implications of DBI_sum_ and DBI_mean_ and suggested a total score calculation for conservation prioritization based on permutation. This algorithm shows the percentile of a community compared to a set of randomly assembled communities of the same species richness. We also present the ‘dragDBI’ package for the statistical software R, a tool for more automated DBI-based environmental health assessments. Our permutational calculation is applicable to other macroinvertebrate-based scoring methods, such as the Biological Monitoring Working Party and the Average Score Per Taxon.

## Introduction

Due to rapid human-mediated habitat loss and fragmentation during the Anthropocene, freshwaters have become the most threatened ecosystems in the world, with many species having declined significantly^1–3^. Therefore, detecting and defining the impacts of habitat modification on freshwater biota is crucial for developing compensatory measures for targeted conservation management^4,5^. Due to limited time, funds, and personnel, practical tools are needed for a rapid and effective assessment of the conservation values of communities^6^. These tools may be based on surrogate taxa that effectively indicate freshwater conditions and can be applied to various freshwater habitats worldwide^5,7^.

Odonates are popular indicators used in environmental health assessments and conservation practices worldwide^8–10^. Due to their biphasic life cycle and high sensitivity to water quality and habitat structure, they readily indicate changes in both freshwater and adjacent terrestrial environments^9^. Adults have diurnal activity, are conspicuous, and can be identified in the field to the species level^11–13^. The Dragonfly Biotic Index (DBI) has become an increasingly popular tool for habitat quality assessments. DBI operates at the species level and is adult-based, sometimes supplemented by larvae and exuviae^14^. Each odonate species within an assemblage is assigned a DBI value that is calculated as the sum of three subindices: geographical distribution, conservation status according to the IUCN Red List, and sensitivity to disturbance^15^. Each sub-index is scored from 0 to 3, giving widespread habitat generalists a score of 0, whereas restricted, sensitive habitat specialists receive the highest cumulative score of 9.

The index was originally developed to measure the ecological integrity of lotic habitats in South Africa^15–17^, and its use has expanded to habitat quality assessments^18^, measuring restoration success, and prioritizing sites for conservation^19^. In Central Europe, DBI has been adopted to evaluate the attractiveness and conservation value of secondary habitats^20–22^. The practical use of the index is currently limited to regions with known species sensitivities, threat levels, and distributions (Africa^4,5,23,24^, and Europe^25–28^, but by raising awareness of these characteristics, it may be easily adapted for use worldwide^19^.

For assessing the conservation value of the site, DBI may be interpreted either per assemblage (i.e., the total score is obtained as the sum of the values of all species in the assemblage, hereafter referred to as DBI_sum_), or per species (i.e., dividing this total by species richness; hereafter referred to as DBI_mean_). Rosset et al.^6^ in their review evaluated the performance of four types of scoring methods for assessing the conservation value of the freshwater habitats, based on the weight given to Red List categories and the total score interpretation (either per assemblage or per species). DBI was one of the metrics included in the comparison, but the authors worked only with DBI_mean_. Since that review, a growing number of studies using DBI have emerged, especially in Central Europe. These studies used both approaches to calculate the total DBI score^20–22,26^. Therefore, the need for clarification for the use of the calculation approach for specific purposes has arisen. Briggs et al.^18^ suggested that using DBI_mean_ is more accurate as it accounts for species richness, including both high-scoring species that raise the average score, and low-scoring species that decrease the average score. However, the presence of generalist species may not inherently indicate a lower habitat quality, integrity, or conservation value, at least not in the conditions of Central Europe^25,29,30^. As a result, the DBI_mean_ applied to the conditions different from those for which it has been developed may be insensitive, as it may be too low for the assemblages with species of higher conservation value when supplemented with widespread generalist species with a score of 0. In contrast, the interpretation of DBI_sum_ does not account for these species, but when many low-scoring species are present at the site, it may favor large communities at the expense of their quality.

In studies using DBI for conservation objectives, there has been a tendency to use DBI_mean_ in comparative assessments^4,14,17,22,23,30–32^. However, certain studies applied DBI_sum_^19–21,24^, or used both approaches^16,18,26,33^ (see Supplementary Information 1). This bifurcation in methodology may stem from specific conditions in various biogeographic regions. In Europe, the proportion of endemic and rare species with high DBI scores is very low^34^, compared with South Africa where the index was developed^35,36^. Moreover, in Europe, there are disturbed habitats hosting rare species alongside many generalists^20,21^. The choice of the total score calculation approach may thus be critical for interpretations in terms of management recommendations.

The same applies to other qualitative scoring methods, such as the Biological Monitoring Working Party (BMWP) that was developed in England in 1976^37^ as a simplified method for lotic water quality assessments using benthic macroinvertebrates^38^. Each family within the assemblage is given a score based on the tolerance to pollution (the greater the tolerance, the lower the score). The total score is calculated by summing the values of all families and order Oligochaeta^39^. The BMWP score may be expressed as the Average Score Per Taxon (ASPT), which is calculated by dividing the BMWP score by the number of families^40^. Since its development, BMWP has been widely used in river health assessments throughout Europe, following the requirements of the Water Framework Directive^41,42^, and its use has rapidly increased for lentic systems as well^43^.

In the present study, we used DBI to evaluate the differences between the approaches to the total score calculation of qualitative scoring methods to help choose the most suitable approach to ecological health assessment. We also tested the new possibility of DBI calculation for practical use in a Central European setting, with a special focus on conservation prioritization. Using a well-defined set of Central European odonate species along simulated scenarios, our objective was to (i) test for the attributes of DBI_sum_ and DBI_mean_ under various conditions (high vs low richness, combined with the presence or absence of high- or low-scoring species), (ii) suggest a unifying method of using DBI for conservation prioritization and compare its outcomes with those of DBI_sum_ and DBI_mean_, and (iii) present a new package in the statistical software R for the unified and more automated DBI-based ecological health assessments. The presented approach may be potentially applicable to other globally and extensively used macroinvertebrate-based scoring methods using qualitative data, such as BMWP (analogous to DBI_sum_) and ASPT (analogous to DBI_mean_).

## Materials and methods

First, we focused on the differences between DBI_sum_ and DBI_mean_. We used a set of Central European odonate species as a model group because out of 73 species, 68 have defined DBI scores (Supplementary Information 2). We used two types of arithmetic average, which we recognized for clarity as (i) the mean of DBI when working with the DBI_mean_ of species within one community (i.e., calculated by summing the DBI scores of all species present in a community and then dividing this total by the number of species), and (ii) the average of DBI values when calculating average DBI_sum_ or DBI_mean_ for the set of communities (i.e., summing either the DBI_sum_ or DBI_mean_ scores for all communities considered in the study and then dividing the resultant total by the number of these communities). All subsequent analyses, in which we assessed the performance of the calculation methods, were performed in R 4.2.1^44^.

### Assessing the performance of DBI calculation methods

**Figure 1:**
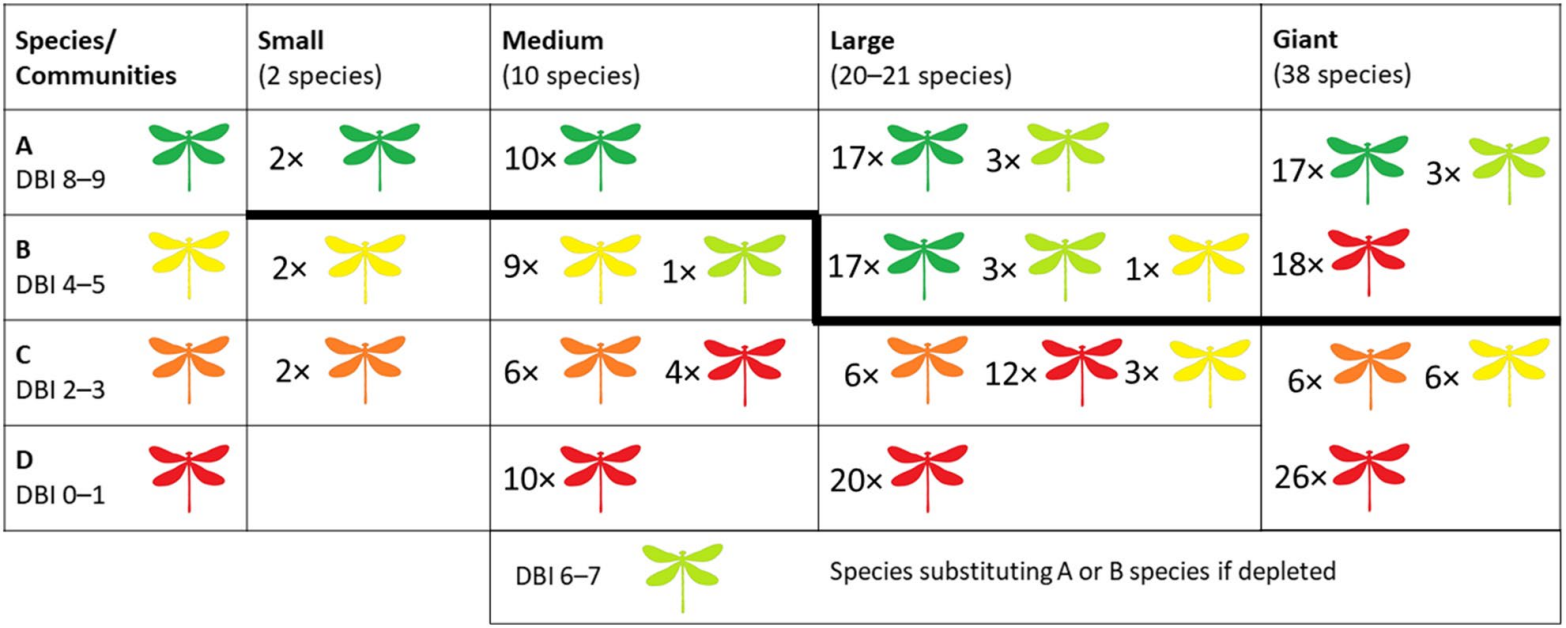
Representation of the 13 simulated scenarios illustrating the composition of species with various DBI values across different community sizes (small, medium, large, and giant). Rows categorize species types based on DBI scores; ‘A’ (DBI = 8–9), ‘B’ (DBI = 4–5), ‘C’ (DBI = 2–3), or ‘D’ (DBI = 0–1), while columns delineate the specific scenarios devised for each community size. Light green species act as substitutes for ‘A’ and ‘B’ species when depleted, necessitating the selection of species with lower DBI values. This is particularly evident when constructing large and giant communities and the depletion of species with the corresponding DBI values occurs, prompting the inclusion of species from the nearest DBI categories. A bold line distinctly separates communities with a prevalence of high-scoring ‘A’ species, indicating scenarios where a shift to a high-scoring species scenario was made at the expense of the scenario that should have been in the given field according to the basic description of the figure.

To examine the attributes of DBI_sum_ and DBI_mean_, we created 13 representative scenarios of simulated communities. These scenarios varied in species richness and community composition (i.e., also DBI values of the species involved). Species richness (i.e., the number of species) was selected based on a literature review (see Supplementary Information 1). We used the (i) median of the lowest richness for small communities, (ii) median of the highest richness for large communities, (iii) overall median for medium communities, and (iv) the maximum richness for ‘giant’ communities. The range of DBI values of species in the scenarios worked with the ‘A’ species (DBI = 8–9), ‘B’ species (DBI = 4–5), ‘C’ species (DBI = 2–3), and ‘D’ species (DBI = 0–1; Fig. 1, Table 1). In large communities, once all the high-scoring species have been recorded at a site and the remaining taxa could only include the species with lower DBI values (hereafter we refer to this situation as “depletion”), the values were supplemented by the closest possible DBI values (e.g., for the ‘A’ species with DBI = 7). For each of the thirteen communities, we calculated DBI_mean_ and DBI_sum_.

**Table 1:** Characteristics of the species used for the simulated scenarios, based on the DBI values of each group (distribution, conservation status, sensitivity to disturbances).

Species	DBI values	Characteristics
A	8–9	Restricted, endangered, extremely sensitive
B	4–5	Rare, vulnerable, fairly sensitive
C	2–3	Common, nearly threatened, weakly sensitive
D	0–1	Very common, not threatened, least sensitive

Subsequently, to develop a straightforward tool for conservation prioritization, we created a function that randomly assembles 10,000 communities with the same species richness as the community of interest, from the whole species pool (here for the Central European pool) without replacement. This ensured that each species was unique within a community, preventing duplication. The number of permutations with a resulting DBI_sum_ lower than the actual DBI_sum_ for the given community is compared to the total number of permutations, giving the permutational DBI potential (for the rationale of its development, see Supplementary Information 3). The permutational DBI potential thus shows the percentile that the community reaches compared to a set of randomly assembled communities with the same species richness. The probability weights of individual DBI values were set as P_DBI_ = x^−DBI^. The formula is based on the presumption that the DBI inherently reflects the varying frequency/rarity and sensitivity of species, and thus the varying probability of individual scenarios. If we consider that communities with the same DBI_sum_ and DBI_mean_ (i.e., the same species richness) should have the same permutational DBI potential, regardless of the combination of species’ DBI values (e.g., community with two species with DBI = 3 and DBI = 4 should be equal to a community with two species with DBI = 0 and DBI = 7), then the probability weights of individual DBI values (P_DBI_) should follow the function y = 1/(x^DBI^) (i.e., x^−DBI^). To estimate parameter x, we focused primarily on the distribution subindex of DBI (DBID). Although each subindex of DBI (i.e., distribution, threat, and sensitivity) should reflect the probability of encounter of the species, distribution should be the most directly linked to the frequency of the occurrence. Considering the number of quadrats occupied by each Central European species in the Czech Republic, we calculated the occupancy rate by relating these counts to the number of quadrats occupied by the most common species (i.e., the species with occupancy rate = 1). We omitted species with DBID = 3, as in the Czech Republic these species occur frequently on the edge of their distribution; moreover, DBID = 3 is highly correlated with the highest values of the two other two subindices (threat and sensitivity), thus confounding the calculation.

To assess the stability of the permutational DBI potential calculation and thus its reliability and applicability in real-world scenarios, we employed data from the study of Šigutová et al.^27^ using the default setting of 10,000 permutations. These data comprise odonate communities in 11 retention ponds and 11 control ponds. We assume that these data effectively represent diverse dragonfly communities in Central Europe (ranging in species richness from 6 to 24 species). For each community, we computed the permutational DBI potential 100 times and generated a frequency distribution function for each set of values. We assessed whether these distributions differ from each other using the set of pairwise two-sample Kolmogorov–Smirnov tests. The resulting p-values were adjusted by Bonferroni correction.

## Results

Simulated communities differed in their ranking based on the total score calculation method. DBI_sum_ ranked a large community composed of ‘C’ species for given hypothetical scenarios higher than a small community composed of ‘A’ species, whereas DBI_mean_ ranked a small or medium community of ‘A’ species better than a large community of ‘A’ species. The values of the permutational DBI potential of high-quality communities were almost equal, regardless of species richness (Table 2).

**Table 2:** Thirteen simulated scenarios worked with giant (38), large (20–21), medium (10), or small (2) communities of ‘A’ (DBI = 8–9), ‘B’ (DBI = 4–5), ‘C’ (DBI = 2–3) or ‘D’ species (DBI = 0–1).

Simulated scenarios	DBI_sum_	DBI_mean_	Permutational DBI potential	DBI_sum_ rank	DBI_mean_ rank	Permutational DBI rank
giant com. (20 ‘A’ sp. + 18 ‘D’ sp.)	163	4.29	1.000	2	5	1
giant com. of ‘D’ sp.	54	1.42	0.000	5	9	7
large com. of ‘A’ sp. + 1 ‘B’ sp.	167	7.95	1.000	1	3	1
large com. of ‘A’ sp.	162	8.10	1.000	3	2	1
large com. of ‘C’ sp.	34	1.70	1.000	7	8	1
large com. of ‘D’ sp.	8	0.40	0.188	11	10	6
medium com. of ‘A’ sp.	85	8.50	1.000	4	1	1
medium com. of ‘B’ sp.	45	4.50	1.000	6	4	1
medium com. of ‘C’ sp.	18	1.80	0.997	9	7	3
medium com. of ‘D’ sp.	4	0.40	0.439	12	10	5
small com. of ‘A’ sp.	17	8.50	1.000	9	1	1
small com. of ‘B’ sp.	9	4.50	0.998	10	4	2
small com. of ‘C’ sp.	4	2.00	0.946	12	6	4

DBI_sum_—the DBI_sum_, DBI_mean_—the DBI_mean_, Permutational DBI potential—percentile for the community DBI compared to a set of randomly assembled communities with the same species richness; DBI_sum_ rank, DBI_mean_ rank, and Permutational DBI Rank—Rank based on DBI_mean_, DBI_sum_, and permutational DBI potential, respectively—from the most to the least valuable community.

To set the parameter x in the probability weight function, we counted the following average occupancy rates: R_DBID = 0_ = 0.80, R_DBID = 1_ = 0.38, R_DBID = 2_ = 0.18, implying that with the increasing DBID, the frequency of the species decreased roughly by half. Due to the lack of quantitative data on which to base our decision regarding subindices of threat and sensitivity, and regarding their same weights in the total DBI value calculation, we adopted the same principle as for DBID. Therefore, each increase in the total DBI of the species leads to half the probability of its occurrence/or the species is twice as sensitive/twice as endangered (that is, x = 2, and therefore the probability weights of the individual DBI values are y = 2^−DBI^). We are aware that this value may not yet accurately reflect the desired final values; however, other settings led to only slightly different, highly correlated results (not presented).

Calculation of permutational DBI potential with the probability weight function y = 2^−DBI^ using real data from Šigutová et al. (2022) showed that from 22 communities, only two (Sto4 and Sto9) could not be reliably discriminated (Supplementary Information 4). In both cases, the permutation DBI potential = 1 for the majority of repetitions. However, DBI_mean_ (Sto4 = 2.44, Sto9 = 1.70) and DBI_sum_ (Sto4 = 22, Sto9 = 34) differed between these two communities due to different species richness (Sto4 = 9 species, Sto9 = 20 species). These results indicate good discrimination between real communities and the stability of the permutation DBI potential when using 10,000 permutations, suggesting the suitability of this approach for conservation practice.

### Novel statistical tool for DBI-based assessments

To facilitate the use of DBI in environmental bioassessments and various DBI-based calculations, and for simple calculation of permutational DBI potential, we created the package ‘dragDBI’ (version 1.0) in statistical software R, available from GitHub at https://github.com/VeronikaPrielozna/dragDBI. The package also serves as a database of the current DBI values for Central European and South African odonate species. All instructions for installation of the current release or development versions can be consulted on the GitHub repository page (https://github.com/VeronikaPrielozna/dragDBI/discussions). Currently, DBI values can be calculated for the Central European Dataset or South African Dataset; however, users can also load their checklists including DBI values using the ‘LoadDBI’ function. The main ‘CalculateDBI’ function calculates the DBI_sum_, DBI_mean_, and permutational DBI potential (and also DBI potential and real DBI potential, see Supplementary Information 3) for odonate community samples used as input, comparing them with DBI-supplied checklists of dragonfly species from Central Europe or South Africa. The ‘dragDBI’ package will be kept up-to-date with new entries in the checklists included in the package.

## Discussion

The Dragonfly Biotic Index, an easy-to-use tool for freshwater health assessments, has gained popularity over recent years^5,20,21,23–27,45,46^. Being originally developed for South African species^15^, its use has expanded to other geographical regions^6,20,22,30,47–49^ (see Supplementary Information 1). The resilience and wide applicability potential of the index can be documented by its extensions for African locations, the Habitat Condition Scale (HCS), which incorporates the structural condition of the habitat^19^, or the Biodiversity Recovery Score (BRS), which is a ratio of the sum of the DBI scores before and after the conservation measure at a particular site^15^. DBI meets the criteria for a practical index used for prioritizing sites or for assessing the success of conservation action as it provides reliable and repeatable results while being sufficiently sensitive as it operates at the species level with a conspicuous and relatively easily identifiable taxon^19,50,51^. Nevertheless, regarding different approaches to calculating DBI and considering its application to conditions different from those for which it has been originally developed, the choice of the total score calculation approach must be carefully considered.

Although certain authors have suggested that the use of DBI should be supplemented by species accumulation curves to ensure that it is calculated based on the full inventory of dragonfly species^18^, the authors of the original concept emphasize the need to consider flight periods of all local species and that the site-specific index encompasses the core resident species, not vagrant species^17^. In addition, they supported using standardized scores (DBI_mean_) for environmental monitoring to compare DBI scores among sites. This approach has been derived from the most standardized measure in freshwater health assessment using macroinvertebrates; the Average Score Per Taxon (ASPT) which accounts for the species richness. The main advantage of ASPT is to suppress the effect of sampling effort and seasonal variation^40^. The use of DBI_mean_ was further recommended, as it provides additional information over species richness, since both approaches are not correlated, unlike other scoring methods calculated as a sum^6^. Consequently, DBI_mean_ has been extensively used in comparative evaluations (Supplementary Information 1).

Nevertheless, for conservation prioritization, especially in the areas without a high level of endemism and a high proportion of rare species, this approach may be unsuitable. According to our simulations, the ranking of communities differed based on the total score calculation approach (DBI_sum_ or DBI_mean_). Although the average DBI_mean_ is equal for any number of species, the variability depends on species richness. While for one species, the DBI_mean_ can range between 0 and 9, with increasing species richness, the variability decreases due to the restriction of the number of species with certain DBI values, and for the whole species pool the DBI_mean_ is determined. Consequently, smaller communities of ‘A’ species (DBI = 8–9) could be ranked better than a larger community of ‘A’ species, especially in large communities where depletion of species with the highest DBI values may occur. Furthermore, if a large community predominantly composed of 'A' species is supplemented with several 'D' species (DBI = 0–1), it leads to a significant decrease in the DBI_mean_, even below the values of smaller communities lacking ‘A’ species, but without ‘D’ species. Similarly, the application of DBI_sum_ may be inappropriate in certain situations. Unlike the average DBI_mean_, the average DBI_sum_ inherently depends on species richness (although it does not account for species with DBI = 0); therefore, species-rich communities composed of species with DBI ≥ 1 can be considered more valuable than small communities of species with higher quality. As a result, the DBI_sum_ of a large community composed of ‘C’ species (DBI = 2–3) could be higher than that of a small community composed of ‘A’ species.

However, a species-rich community may not always be a valuable community, because species richness itself is a problematic indicator of quality. Following environmental changes, species composition may change but richness may remain unchanged^14,19,20^, or may increase following disturbance^52^. Moreover, richness depends heavily on the extent of sampling effort^53,54^ and cannot be compared against an absolute standard^55^. Furthermore, it varies across different habitat types^56,57^. Therefore, based on the illustration of the range of values that DBI (DBI_sum_ or DBI_mean_ indiscriminately) can take for a given species richness, we calculated the permutational DBI potential that relates the DBI of the community to the potential values for the set of random communities. This approach combines the advantages of DBI_mean_ and DBI_sum_ while addressing the differences in their interpretation. Similar to DBI_mean_, the permutational DBI potential is not constrained by the species richness per se. At the same time, it is robust towards supplementing high-quality communities with less valuable species, thereby solving the main issue that may arise if DBI_mean_ is misinterpreted. Adding low-scoring species to a top-quality community (when high-scoring species are depleted) does not decrease the permutational DBI potential (unlike DBI_mean_). Therefore, for conservation prioritization, counting permutational DBI potential seems to be the optimized solution. The zero chance that the community could be assembled in the same way or better (i.e., permutational potential = 1) will arise for (i) small communities only of ‘A’ species, (ii) medium communities only of ‘A’ or ‘B’ species, and (iii) large communities of ‘A’, ‘B’, and ‘C’ species. From a conservation point of view, all these communities would be equally valuable.

In our simulations, seven of 13 scenarios ended up at the maximum permutational DBI potential (1.00), suggesting that its use may seem excessively stable or insensitive. The maximum permutation DBI potential may also arise for a large community of ‘A’ species, even when supplemented by ‘D’ species. The reason is that communities may be relatively far from their potential DBI_mean_ or DBI_sum_ maximum (for a given number of species), but the number of communities that could fit between the real and the potential maximum DBI is combinatorically minimal because high-scoring species are inherently less frequent. To simulate this unequal distribution of high- and low-scoring species within natural communities, we used 2^−DBI^ as a probability weight function. This setting proved to be the most suitable during the testing using the trial dataset. For this setting, permutational DBI potential may penalize large communities supplemented by ‘D’ species, but only if the pool of species with higher DBI values is not depleted, and, simultaneously, the pool of supplemented ‘D’ species is enormous.

The second problem would potentially arise for giant communities (≥ 32 species) composed of species with the lowest possible DBI values. In such rich communities, when considering the lowest possible DBI values of all species, at least some species would have DBI ≥ 4, yet this community would receive the lowest rating. However, real communities are never exclusively composed of ‘A’ or ‘D’ species, especially the rich ones. In our scenarios, we worked with extreme examples to demonstrate the limits of the approaches to the total score calculation. By exploring these extreme scenarios, we aimed to preemptively address any potential misinterpretations in real-world conservation planning, ensuring the index application remains robust and reliable. Furthermore, considering unlikely scenarios reflects the fact that conservation priorities should focus on top-quality habitats^58^ as the resources available for biodiversity conservation are limited^59–61^. The permutational DBI potential fulfills this requirement.

Apart from its utility for conservation prioritization, using permutational DBI potential would enable meta-analyses of studies using DBI; similar to DBI_mean_, yet unlike DBI_sum_, it is robust to changes in sampling effort. Furthermore, the permutational DBI potential may facilitate cross-regional comparisons where DBI values may be set differently, an aspect not adequately addressed by either DBI_mean_ or DBI_sum_. This distinctive capability enhances the utility of the permutational DBI potential for meta-analyses of studies using DBI across various geographical contexts, allowing for more accurate comparisons across sites, countries, and continents. Additionally, the permutational approach may apply to all systems working on a similar principle. It may be transferred to other qualitative scoring methods, such as Biological Monitoring Working Party Score System (BMWP)^38^, and potentially also to other systems working with a different range of values (e.g., Odonata Index of Wetland Integrity—OIWI^12^, or South African Scoring System—SASS^62,63^). However, before this application can be generalized, additional testing on real communities or simulations is required to establish the parameters x for the probability weight function.

From the development of permutational DBI potential, the need for an automated tool for its computation arose. Therefore, we created a package in the statistical software R to enable the calculation of both permutational DBI potential and other DBI scores. Another objective was to create a database of the available DBI values, making them easily accessible for conservation practice and enabling further analyses with these scores. Currently, the ‘dragDBI’ package provides biomonitoring practitioners with a reliable and up-to-date open-source tool for transparent DBI computation from presence/absence or abundance data. It also enables users to upload their own species data for index calculation. Our package may be used not only for direct assessments of the conservation values of the sites (by calculating DBI_mean_, DBI_sum_, and permutational DBI potential), but also as a source of DBI values for calculations of various indices using DBI as a core metric, such as Index of Summed Rarity^27,64^, or abundance-weighted DBI ^26^.Therefore, with accessible DBI values, our package may be a good starting point for a wider application of the index. Furthermore, our algorithm, along with the functions created within the package, will allow future additions of parallel solutions for other qualitative scoring systems, after appropriate adjustment of the x parameter for the probability weight function.

## Conclusions

We show that qualitative scoring metrics, such as DBI, may have different interpretations for conservation practice depending on the approach to the total score (expressed as a sum or mean). The permutational solution seems to be the most promising for conservation prioritization, being robust and sufficiently sensitive to the specificities of the index. In summary, permutational DBI potential may be a powerful tool for freshwater conservation. Sampling odonate adults, which have been shown to perform well for DBI comparisons^31^, provides a considerable advantage over traditional labor-intensive and challenging macroinvertebrate-based surveys^6,19^. Samples can be identified visually in the field, often using close-focus binoculars^46,65^, thus avoiding community disturbance and lethal sampling. Identification could even be performed by trained non-professionals. Although the use of the index is currently limited to parts of Africa and Europe, expanding knowledge of species sensitivities, threat levels, and distributions, especially in the Neotropics, will soon make species scores readily available to conservation practitioners without individual species assessments. The use of canned information within the ‘dragDBI’ package, which we plan to keep updated, may greatly facilitate the wider application of the Dragonfly Biotic Index in conservation practice.

### Supplementary Information

Supplementary Information 1.

Supplementary Information 2.

## Data Availability

Data (DBI values of individual Central European and South African Species) are permanently archived in the figshare repository (10.6084/m9.figshare.21644621). The novel code (as a part of the ‘dragDBI’ statistical package) is provided via the Github repository (https://github.com/VeronikaPrielozna/dragDBI).
