# Supplementary material for: Sum or mean in calculation of qualitative scoring methods using the Dragonfly Biotic Index, and an alternative approach facilitating conservation prioritization
Source: Sci Rep. 2024 May 18;14:11356. doi: 10.1038/s41598-024-62017-y (PMC11102514; doi:10.1038/s41598-024-62017-y)
Supplement: Supplementary file 1 — Supplementary Information 1. [file 41598_2024_62017_MOESM1_ESM.pdf]

Supplementary Information for Sum or mean in calculation of qualitative scoring methods using the Dragonfly Biotic Index, and an alternative approach facilitating conservation prioritization

Authors: Hana Šigutová, Petr Pyszko, Eva Bílková, Veronika Prieložná, Aleš Dolný

This file includes:

- Supplementary Information 1 (*Review of calculation methods in studies using DBI)*
- Supplementary Information 2 (*Set of Central European odonate species and their DBI values*)
- Supplementary Information 3 (*Rationale behind the unification of DBImean and DBIsum via permutational DBI potential*)

Supplementary Information 1 – Review of calculation methods in studies using DBI

Methods

We used the online databases Scopus[1], Web of Science[2], and Google Scholar to find papers using DBI for comparative environmental health assessments. We used the search string "(Odonata OR dragonfl* OR damselfl*) AND (Dragonfly Biotic Index OR DBI)" under "Article title, Keywords, Abstract" in Scopus, and "Topic" in Web of Science. Subsequently, we examined the records and selected the studies using DBI for comparison of assemblages among habitats. We gathered 59 studies containing the DBI index. Subsequently, we excluded all theoretical studies that relied solely on mentioning DBI values of species in the table, resulting in the exclusion of a total of 22 papers. For further analyses, we determined the method used for DBI calculation (DBI_{sum} or DBI_{mean}) in each study. Studies calculating with DBI in any other way (e.g., weighting DBI values by species abundances) were excluded from the analysis. When available, we also reported number of species to get an idea of the span of the species richness in real studies using DBI. To test whether the DBI_{sum} or the DBI_{mean} is more commonly used and whether there are some differences in their use between Europe and Africa, we analyzed the data with the chi-square test in R 4.2.1[3].

Results

DBI_{mean} has been used in the studies more often than DBI_{sum}, although this difference was not significant (df = 1, χ^2 = 1.60, P = 0.206). Within the studies, only DBI_{mean} was used 19 times, only the DBI_{sum} was used 11 times, and five studies used both approaches. The countries that most often use DBI can be found in Africa and Central Europe (Fig. S1a). Although the trend is not significant, there is a tendency in Europe to use DBI_{sum}, while in Africa, DBI_{mean} prevails (df = 1, χ^2 = 0.96, P = 0.326, Fig. S1b). The range of species richness that is worked with reaches a median of between 2–20 species within one study, if it comprises multiple sites (Fig. S2). Summary of studies used for the analysis is given in Table S1.

---

[1] Elsevier, 2022. Scopus [WWW Document]. https://www.scopus.com (accessed 17 August 2022).
[2] Clarivate, 2022. Web of Science [WWW Document]. https://webofknowledge.com (accessed 15 August 2022).
[3] R Core Team, 2021. R: a language and environment for statistical computing. R Foundation for Statistical Computing, Vienna, Austria. https://www.R-project.org.

Fig S1. (a) Number of studies working with DBI_{sum} and DBI_{mean} by countries; *° More countries processed within one study; (b) Use of DBI_{sum} and DBI_{mean} in Africa and Europe (ratio ± 95% CI)

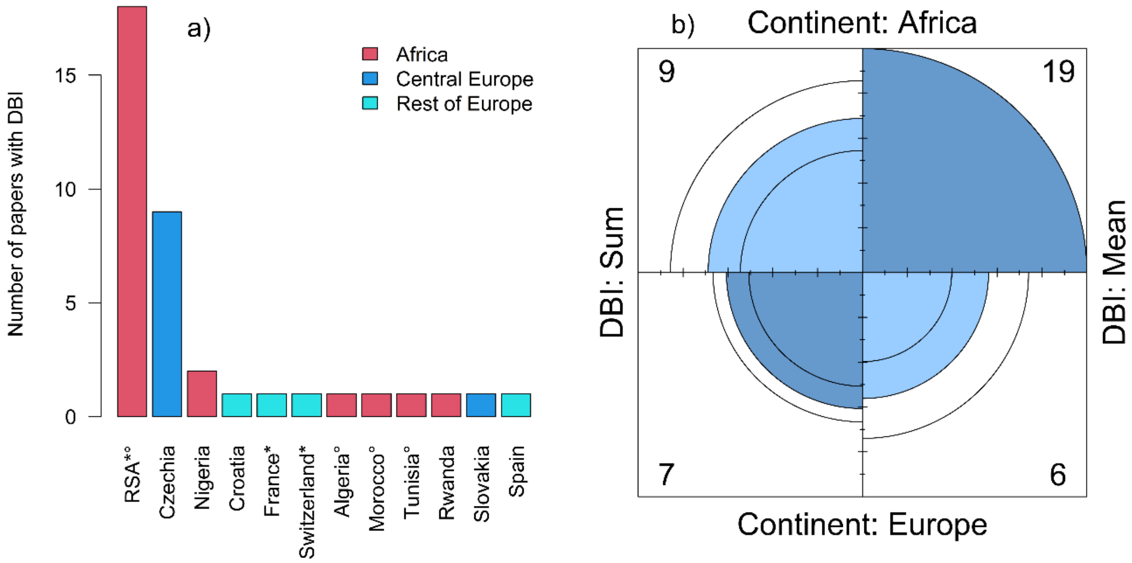

Fig. S2. Range of species richness in studies comparing multiple sites. Median minimum = 2, median maximum = 20

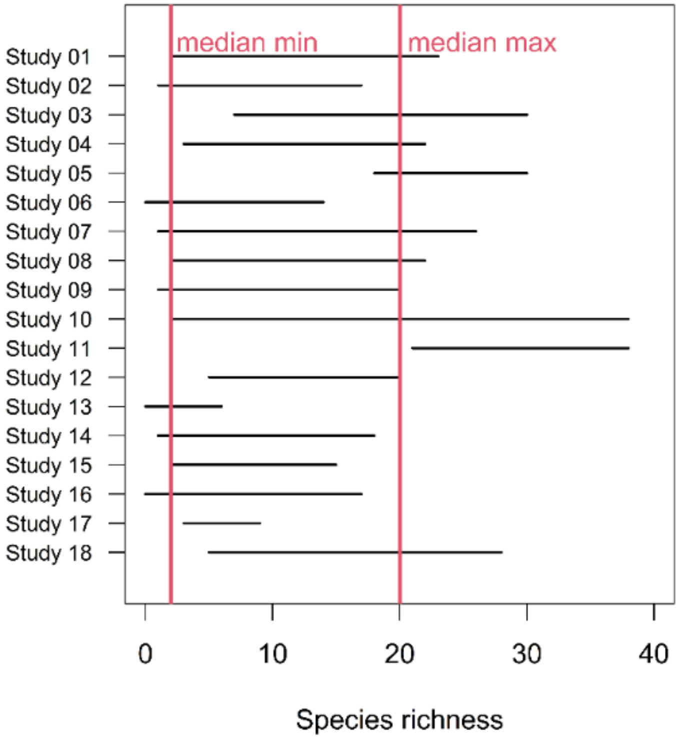

Table S1. Summary of studies using DBI for comparative assessments

Geographical location	Water-body type	Main assessment objective	DBI calculation method	Species richness	Reference
South Africa/ RSA	Rivers	Habitat recovery, habitat quality, conservation prioritization	Mean	NA	Simaika & Samways, 2009
South Africa/ RSA	Rivers	Conservation prioritization, habitat quality	Mean	NA	Simaika & Samways, 2009
South Africa/ RSA	Rivers	Habitat quality	Mean	0–17	Kietzka et al., 2021a
South Africa/ RSA	Rivers	Habitat quality, freshwater diversity	Mean, Sum	2–15	Simaika & Samways, 2011
South Africa/ RSA	Rivers	Conservation prioritization, habitat quality, disturbance	Mean	1–20	Kietzka et al., 2018
South Africa/ RSA	Ponds	Habitat quality	Mean	0–10	Briggs et al., 2019
South Africa/ RSA	Rivers	Habitat quality	Mean, Sum	NA	Grant & Samways, 2011
South Africa/ RSA	Lentic habitats	Conservation prioritization, habitat quality	Mean	NA	Simaika et al., 2016
South Africa/ RSA	Various lotic and lentic habitats	Habitat quality	Sum	0–6	Kietzka et al., 2015
South Africa/ RSA	Rivers, streams	Habitat quality, habitat recovery	Sum	5–20	Simaika & Samways, 2008
South Africa/ RSA	Rivers	Habitat quality, ecosystem integrity	Mean, Sum	0–14	Kietzka et al., 2021b
South Africa/ RSA	Various lotic and lentic habitats	Conservation prioritization, habitat quality	Mean, Sum	2–22	Hart et al., 2014
South Africa/ RSA	Rivers	Habitat quality, disturbance, ecosystem integrity	Mean	NA	Kietzka, 2016
South Africa/ RSA	Lentic habitats	Conservation prioritization, habitat quality	Mean	NA	Simaika et al., 2016
South Africa/ RSA	Lentic habitats	Habitat quality	Mean	1–17	Avidon, 2021
South Africa/ RSA	Wetlands	Habitat quality	Mean	3–9	Kubheka, 2017

Geographical location	Water-body type	Main assessment objective	DBI calculation method	Species richness	Reference
South Africa/ RSA	Ponds	Conservation prioritization, habitat quality	Mean	NA	Briggs, 2016
South Africa/ Swaziland	Various lotic and lentic habitats	Habitat quality, habitat disturbance	Mean, Sum	7–30	Diedericks et al., 2013
North Africa, South Africa/ RSA, Morroco, Algeria, Tunisia	Various lotic and lentic habitats	Conservation prioritization, habitat quality	Mean	NA	Khelifa, 2021
West Africa/ Nigeria	Various lotic and lentic habitats	Habitat disturbance	Sum	NA	Adu et al., 2016
West Africa/ Nigeria	Rivers	Habitat quality	Mean, Sum	18–30	Adu et al., 2019
Central Africa/ Rwanda	Various lotic and lentic habitats	Habitat quality	Mean	NA	Uyizeye, 2020
South Africa, West Europe, Central Europe/ South Africa, France, Switzerland	Ponds, lakes	Habitat quality, conservation value	Mean	NA	Rosset et al., 2013
Southwestern Europe/ Spain	Lentic artificial waterbodies	Habitat quality	Sum	2–23	Monzó, 2015
Southeast Europe/ Croatia	Rivers	Habitat quality	Mean	NA	Vilenica et al., 2022
Central Europe/Czechia	Drainage ditches	Habitat quality, ecosystem integrity	Sum	≤ 10	Tichánek, 2013
Central Europe/Czechia	Mine subsidence pools	Habitat disturbance	Sum	5–28	Harabiš & Dolný, 2015
Central Europe/Czechia	Mine subsidence pools	Habitat quality, freshwater diversity	Mean	1–18	Harabiš et al., 2013
Central Europe/Czechia	Mine subsidence pools	Habitat disturbance, habitat quality	Sum	21–38	Harabiš & Dolný, 2012
Central Europe/Czechia	Mine subsidence pools	Conservation prioritization, habitat quality	Mean	2–38	Dolný & Hatabiš, 2012
Central Europe/Czechia	Lentic habitats	Conservation prioritization, habitat quality	Mean	≤ 30	Harabiš & Dolný, 2018
Central Europe/ Slovakia	Lentic artificial waterbodies	Conservation prioritization, habitat quality	Sum	1–26	Balász et al., 2022

Geographical location	Water-body type	Main assessment objective	DBI calculation method	Species richness	Reference
Central Europe/Czechia	Mine subsidence pools	Habitat quality, conservation value	Mean	3–22	Harabiš, 2016
Central Europe/Czechia	Various lotic and lentic habitats	Habitat quality, conservation value	Sum	NA	Dolný et al. 2021
Central Europe/Czechia	Lentic artificial waterbodies	Habitat quality	Sum	NA	Kolar et al., 2021
Central Europe/ Slovakia	Lentic habitats	Habitat quality	Mean	NA	Petrovičová et al., 2021

References

Adu, B. W., Amusan, B. O., & Oke, T. O. (2019). Assessment of the water quality and Odonata assemblages in three waterbodies in Ilara-Mokin, south-western Nigeria. *International Journal of Odonatology*, 22(2), 101-114.

Adu, B. W., Kehinde, K. K., & Ogbogu, S. S. (2016). Monitoring of environmental disturbance using abundance and distribution of red-vein and dark-vein species of genus Trithemis (Odonata: Libellulidae). *Zoologist (The)*, 14, 31-36.

Avidon, S. N. (2021). Contextualizing waterscape health in a subtropical rangeland (Master thesis, Stellenbosch University).

Balázs, A., Šipoš, J., Matúšová, Z., Hamerlík, L., Novikmec, M., & Svitok, M. (2022). Comparison of conservation values among man-made aquatic habitats using Odonata communities in Slovakia. *Biologia*, 1-13.

Briggs, A. J. (2016). Pond biodiversity in a sugarcane-forestry mosaic in KZN (Doctoral dissertation, Stellenbosch University).

Briggs, A. J., Pryke, J. S., Samways, M. J., & Conlong, D. E. (2019). Complementarity among dragonflies across a pondscape in a rural landscape mosaic. *Insect Conservation and Diversity*, 12(3), 241-250.

Diedericks, G., Simaika, J., & Roux, F. (2013). A Survey of Adult Odonata Along the Crocodile-Inkomati River Main Stem from Source to Ocean. *Report to the Mpumalanga parks and tourism Agency*, 1-56.

Dolný, A., & Harabiš, F. (2012). Underground mining can contribute to freshwater biodiversity conservation: allogenic succession forms suitable habitats for dragonflies. *Biological Conservation*, 145(1), 109-117.

Dolný, A., Ožana, S., Burda, M., & Harabiš, F. (2021). Effects of landscape patterns and their changes to species richness, species composition, and the conservation value of odonates (Insecta). *Insects*, 12(6), 478.

Grant, P. B., & Samways, M. J. (2011). Micro-hotspot determination and buffer zone value for Odonata in a globally significant biosphere reserve. *Biological Conservation*, 144(2), 772-781.

Harabiš, F. (2016). High diversity of odonates in post-mining areas: Meta-analysis uncovers potential pitfalls associated with the formation and management of valuable habitats. *Ecological Engineering*, 90, 438-446.

Harabiš, F., & Dolný, A. (2012). Human altered ecosystems: suitable habitats as well as ecological traps for dragonflies (Odonata): the matter of scale. *Journal of Insect Conservation*, 16(1), 121-130.

Harabiš, F., & Dolný, A. (2015). Odonates need natural disturbances: how human-induced dynamics affect the diversity of dragonfly assemblages. *Freshwater Science*, 34(3), 1050-1057.

Harabiš, F., & Dolný, A. (2018). Military training areas as refuges for threatened dragonfly species: Effect of spatial isolation and military activity. *Biological Conservation*, 217, 28-35.

Harabiš, F., Tichanek, F., & Tropek, R. (2013). Dragonflies of freshwater pools in lignite spoil heaps: Restoration management, habitat structure and conservation value. *Ecological Engineering*, 55, 51-61.

Hart, L. A., Bowker, M. B., Tarboton, W., & Downs, C. T. (2014). Species composition, distribution and habitat types of Odonata in the iSimangaliso Wetland Park, KwaZulu-Natal, South Africa and the associated conservation implications. *PLoS One*, 9(3).

Khelifa, R. (2019). Sensitivity of biodiversity indices to life history stage, habitat type and landscape in Odonata community. *Biological Conservation*, 237, 63-69.

Kietzka, G. J. (2016). The natural drivers and the effects of landscape transformation for dragonflies of the Cape Floristic Region (Doctoral dissertation, Stellenbosch University).

Kietzka, G. J., Pryke, J. S., & Samways, M. J. (2015). Landscape ecological networks are successful in supporting a diverse dragonfly assemblage. *Insect Conservation and Diversity*, 8(3), 229-237.

Kietzka, G. J., Pryke, J. S., & Samways, M. J. (2018). Comparative effects of urban and agricultural land transformation on Odonata assemblages in a biodiversity hotspot. *Basic and Applied Ecology*, 33, 89-98.

Kietzka, G. J., Pryke, J. S., Gaigher, R., & Samways, M. J. (2021a). Congruency between adult male dragonflies and their larvae in river systems is relative to spatial grain. *Ecological Indicators*, 124.

Kietzka, G. J., Pryke, J. S., Gaigher, R., & Samways, M. J. (2021b). Webs of well-designed conservation corridors maintain river ecosystem integrity and biodiversity in plantation mosaics. *Biological Conservation*, 254.

Kolar, V., Vlašánek, P., & Boukal, D. S. (2021). The influence of successional stage on local odonate communities in man-made standing waters. *Ecological Engineering*, 173, 106440.

Kubheka, P. S. (2017). Developing and testing the congruency of selected biological indicators and an existing tool designed to assess wetland health in agricultural setting in the KZN midlands (Master thesis, Stellenbosch University)

Monzó, E. S. (2016). Estructura de comunidades de Odonata en sistemas mediterráneos (Doctoral dissertation, Universitat de València).

Petrovičová, K., Langraf, V., David, S., Krumpálová, Z., & Schlarmannová, J. (2021). Distinct Odonata assemblage variations in lentic reservoirs in Slovakia (Central Europe). *Biologia*, 76, 3727-3741.

Rosset, V., Simaika, J. P., Arthaud, F., Bornette, G., Vallod, D., Samways, M. J., & Oertli, B. (2013). Comparative assessment of scoring methods to evaluate the conservation value of pond and small lake biodiversity. *Aquatic Conservation: Marine and Freshwater Ecosystems*, 23(1), 23-36.

Simaika, J. P., & Samways, M. J. (2008). Valuing dragonflies as service providers. In A. Córdoba-Aguilar (Ed.), *Dragonflies and Damselflies: Model Organisms for Ecological and Evolutionary Research*. Oxford University Press, 109-123.

Simaika, J. P., & Samways, M. J. (2009). An easy-to-use index of ecological integrity for prioritizing freshwater sites and for assessing habitat quality. *Biodiversity and Conservation*, 18(5), 1171-1185.

Simaika, J. P., & Samways, M. J. (2009). Reserve selection using Red Listed taxa in three global biodiversity hotspots: dragonflies in South Africa. *Biological Conservation*, 142(3), 638-651.

Simaika, J. P., & Samways, M. J. (2011). Comparative assessment of indices of freshwater habitat conditions using different invertebrate taxon sets. *Ecological Indicators*, 11(2), 370-378.

Simaika, J. P., Samways, M. J., & Frenzel, P. P. (2016). Artificial ponds increase local dragonfly diversity in a global biodiversity hotspot. *Biodiversity and conservation*, 25(10), 1921-1935.

Simaika, J. P., Samways, M. J., & Frenzel, P. P. (2016). Artificial ponds increase local dragonfly diversity in a global biodiversity hotspot. *Biodiversity and conservation*, 25(10), 1921-1935.

Tichánek, F., & Tropek, R. (2013). Společenstva vážek odvodňovacích kanálů Radovesické výsypky (Bachelor thesis, University of South Bohemia).

Uyizeye, E. (2020). Developing an Odonate-Based Index for Monitoring Freshwater Ecosystems in Rwanda: Towards Linking Policy to Practice through Integrated and Adaptive Management (Doctoral dissertation, Antioch University).

Vilenica, M., Rebrina, F., Ružanović, L., Gulin, V., & Brigić, A. (2022). Odonata Assemblages as a Tool to Assess the Conservation Value of Intermittent Rivers in the Mediterranean. *Insects*, 13(7), 584.

Supplementary Information 2 – Set of Central European odonate species and their DBI values

Table S2. Values of the Dragonfly Biotic Index (DBI) for Czech species as defined by Harabiš and Dolný (2010)[4]. DBI of each species is calculated as the sum of three subindices: geographical distribution, conservation status according to the IUCN Red List (threat), and sensitivity to disturbance[5]. For detailed description of how the values for the Czech species have been obtained, see Table S3

**Species**	**Distribution**	**Threat**	**Sensitivity**	**DBI**
Aeshna affinis	2	2	1	**5**
Aeshna caerulea	3	3	3	**9**
Aeshna cyanea	0	0	0	**0**
Aeshna grandis	0	0	1	**1**
Aeshna isoceles	2	2	2	**6**
Aeshna juncea	2	1	2	**5**
Aeshna mixta	0	0	1	**1**
Aeshna subarctica	3	3	3	**9**
Anax ephippiger	NA	NA	NA	**NA**
Anax imperator	0	0	0	**0**
Anax parthenope	1	2	0	**3**
Brachytron pratense	2	3	2	**7**
Calopteryx splendens	0	0	0	**0**
Calopteryx virgo	0	0	1	**1**
Coenagrion hastulatum	1	1	2	**4**
Coenagrion lunulatum	3	3	3	**9**
Coenagrion ornatum	2	3	3	**8**
Coenagrion puella	0	0	0	**0**
Coenagrion pulchellum	1	0	1	**2**
Coenagrion scitulum	3	3	1	**7**
Cordulegaster bidentata	2	2	2	**6**
Cordulegaster boltonii	1	2	2	**5**
Cordulegaster heros	NA	NA	NA	**NA**
Cordulia aenea	0	0	0	**0**
Crocothemis erythraea	1	0	0	**1**
Enallagma cyathigerum	0	0	0	**0**
Epitheca bimaculata	3	3	2	**8**
Erythromma lindenii	NA	NA	NA	**NA**
Erythromma najas	0	0	1	**1**
Erythromma viridulum	1	1	1	**3**

[4] Harabiš, F., and A. Dolný. 2010. Využití vážek jako environmentálních indikátorů. Pages 109–116 *in* A. Dolný and F. Harabiš, editors. Vážky 2010. Sborník referátů XIII. celostátního semináře odonatologů v Podyjí. ZO ČSOP, Vlašim

[5] Simaika, J. P., & Samways, M. J. (2008). Valuing dragonflies as service providers. In A. Córdoba-Aguilar (Ed.), *Dragonflies and Damselflies: Model Organisms for Ecological and Evolutionary Research* (pp. 109–123). Oxford University Press.

Gomphus pulchellus	NA	NA	NA	**NA**
Gomphus vulgatissimus	1	2	1	**4**
Chalcolestes viridis	0	0	1	**1**
Ischnura elegans	0	0	0	**0**
Ischnura pumilio	0	1	0	**1**
Lestes barbarus	1	2	2	**5**
Lestes dryas	1	2	2	**5**
Lestes macrostigma	3	3	2	**8**
Lestes sponsa	0	0	0	**0**
Lestes virens	1	2	1	**4**
Leucorrhinia albifrons	3	3	3	**9**
Leucorrhinia dubia	2	2	3	**7**
Leucorrhinia pectoralis	1	2	3	**6**
Leucorrhinia rubicunda	2	3	3	**8**
Libellula depressa	0	0	0	**0**
Libellula fulva	3	3	2	**8**
Libellula quadrimaculata	0	0	0	**0**
Nehalennia speciosa	3	3	3	**9**
Onychogomphus forcipatus	2	3	2	**7**
Ophiogomphus cecilia	1	3	2	**6**
Orthetrum albistylum	1	0	1	**2**
Orthetrum brunneum	2	3	2	**7**
Orthetrum cancellatum	0	0	0	**0**
Orthetrum coerulescens	2	3	3	**8**
Platycnemis pennipes	0	0	0	**0**
Pyrrhosoma nymphula	0	0	0	**0**
Somatochlora alpestris	2	3	3	**8**
Somatochlora arctica	2	3	3	**8**
Somatochlora flavomaculata	2	3	2	**7**
Somatochlora meridionalis	NA	NA	NA	**NA**
Somatochlora metallica	0	0	0	**0**
Stylurus flavipes	2	3	2	**7**
Sympecma fusca	0	0	1	**1**
Sympecma paedisca	3	3	2	**8**
Sympetrum danae	0	0	1	**1**
Sympetrum depressiusculum	2	3	3	**8**
Sympetrum flaveolum	0	0	0	**0**
Sympetrum fonscolombii	2	3	2	**7**
Sympetrum meridionale	2	3	2	**8**
Sympetrum pedemontanum	2	3	3	**8**
Sympetrum sanguineum	0	0	0	**0**
Sympetrum striolatum	1	1	1	**3**
Sympetrum vulgatum	0	0	0	**0**

Table S3. Categories used in Harabiš and Dolný (2010) for establishing DBI values of the Czech odonate species

SCORE	DISTRIBUTION [6]	THREAT [7]	SENSITIVITY [8]
0	Very common throughout the Czech Republic	LC	Not sensitive to or profiting from habitat disturbance
1	Common, rare in certain regions	NT	Low sensitivity to disturbance
2	Rare throughout the Czech Republic	VU	Medium sensitivity to disturbance
3	A few locations throughout the Czech Republic	EN, CR	Extremely sensitive to disturbance

[6] Distribution data from Species Occurrence Database, Nature Conservation Agency of the Czech Republic

[7] National Red List of Threatened species; Hanel L., Dolný A. & Zelený J. 2005: Odonata (vážky). pp. 125-127. In: Farkač J., Král D., & Škorpík M. (eds.): Červený seznam ohrožených druhů České republiky. Bezobratlí. Agentura ochrany přírody a krajiny ČR, Praha, 760 pp

[8] An expert assessment derived from an examination of the species ecological context conducted by the authors.

Supplemental Information 3 – Rationale behind the unification of DBI_{mean} and DBI_{sum} via permutational DBI potential

If we calculate the DBI_{mean} for the whole Central European species pool, the DBI_{mean} = 4.11765. Therefore, the average DBI_{sum} for 1–68 species has a uniformly increasing function, where the average DBI_{sum} = 4.11765 × Species richness. If we further calculate the maximum and minimum possible DBI_{sum} for 1–68 species (by adding species in the descending or ascending order of DBI, respectively), the maximum and minimum DBI_{sum} for a given number of species have both nondecreasing functions intersecting for the full set of species (Fig. S3a) and unambiguously defining the limits within which the values can oscillate.

Unlike the average DBI_{sum}, the average DBI_{mean} remains the same for 1 as well as 68 species; however, the range of possible values the DBI_{mean} varies with species richness. For communities of one species, the DBI_{mean} can range between 0 and 9, but for the full set of species it is clearly given in advance. Thus, as species richness increases, the variance of the DBI_{mean} decreases (Fig. S3b).

Fig. S3. (a) Average DBI_{sum} and (b) average DBI_{mean} (white), the maximum $DBI_{sum/mean}$ (red), and the minimum $DBI_{sum/mean}$ (green) for given species richness of community.

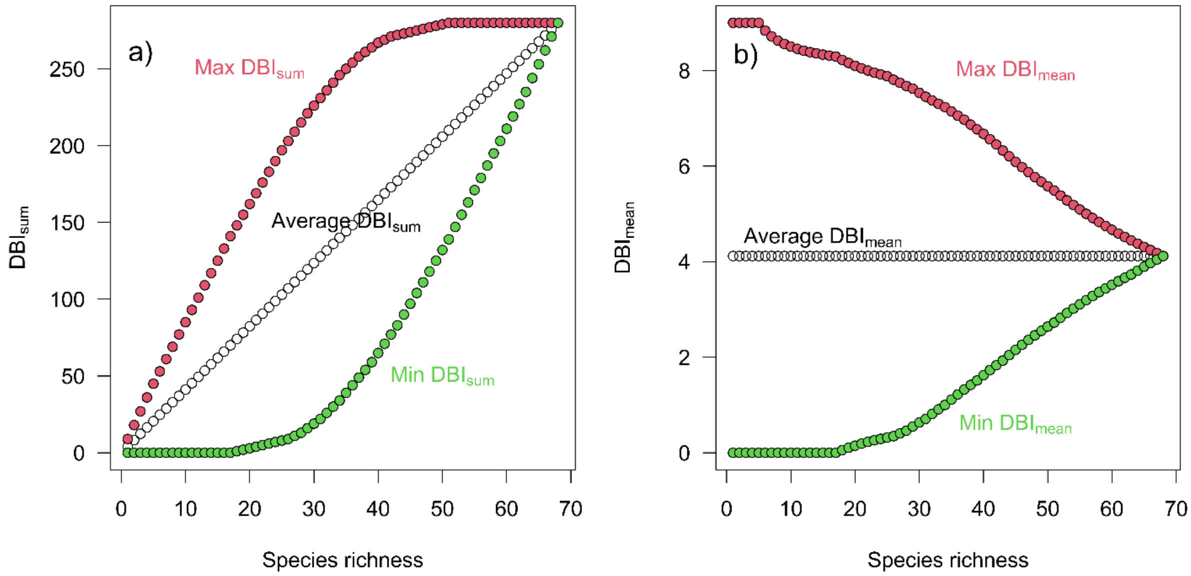

Subsequently, for 1–68 species, we can calculate the actual position of the average DBI_{sum} between the maximum and minimum for a given number of species using the formula: $y_n = (\bar{x}DBI_n - minDBI_n)/(maxDBI_n - minDBI_n)$, where n is the species richness between 1–68, $\bar{x}$ is the average DBI_{sum} for a given number of species, max and min are the maximum and minimum DBI_{sum} for a given number of species, and y is referred to as the **real DBI potential**. We can also calculate simply only the ratio between the average DBI_{sum} and the maximum DBI_{sum} (hereafter called the **DBI potential)**. A parallel procedure can be used to display the average DBI_{mean} for 1–68 species, the maximum DBI_{mean}, and the minimum DBI_{mean} achievable for 1–68 species, and to calculate their DBI potential and the real DBI potential.

The courses of the curves in both cases (for DBI_{sum} and DBI_{mean}) show that the average values can only move within certain limits defined by species richness. The average is approximately in the middle of the interval between the minimum and maximum. However, the uneven number of species in individual DBI classes makes this function neither uniform nor always increasing, although the overall trend is

slowly rising. Therefore, the values (for DBI_{sum} or DBI_{mean}) for the specific community can be related to the potential maximum and minimum (**real DBI potential**). To simplify the matter, the values of DBI (again for DBI_{sum} and DBI_{mean}) for the specific community can be related only to maximum (**DBI potential).** The results are identical, regardless of whether DBI_{sum} or DBI_{mean} are used for the calculation (Fig. S4a, S4b).

Fig S4. Average (a) DBI_{sum} and (b) DBI_{mean} for given species richness related to the interval between minimum and maximum $DBI_{sum/mean}$ (white), and only to the maximum $DBI_{sum/mean}$ (red), and the relationship between maximum and minimum $DBI_{sum/mean}$ (green)

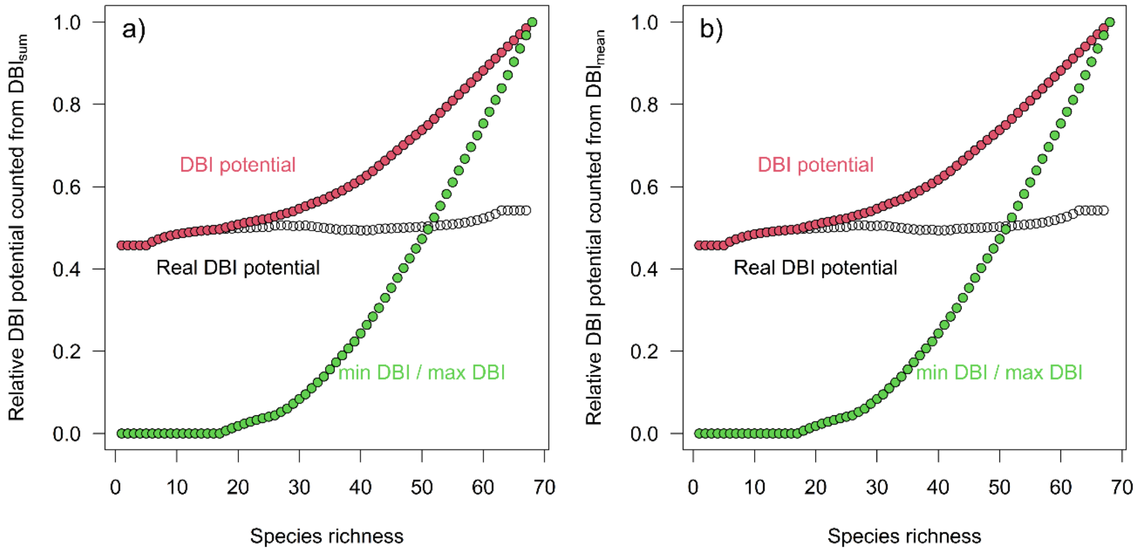

Neither the DBI potential nor the real DBI potential reflect the unequal density of DBI scenarios between minimum and maximum. If species with different DBI values had the same probability of selection, there would be many possibilities to assemble the community around the average value, and the number of possibilities would decrease toward the maximum and minimum. However, the DBI inherently reflects the varying frequency/rarity and sensitivity of species, and thus the varying probability of individual scenarios. If we consider that communities with the same DBI_{sum} and DBI_{mean} (i.e., the same species richness) should have the same permutational DBI potential, regardless of the combination of species' DBI values (e.g., community with two species with DBI = 3 and DBI = 4 should be equal to a community with two species with DBI = 0 and DBI = 7), then the probability weights of individual DBI values (P_{DBI}) should follow the function $y = 1/(x^{DBI})$ (i.e., x^{-DBI}). Based on our calculations, x was set to 2 (for details, see the main body of the manuscript). In Fig. S5 we can see the results of 10,000 permutations for each level of species richness performed with probability of selection of individual species set by this formula.

Fig. S5. Set of possible (a) DBI_{sum}, (b) DBI_{mean} for randomly simulated communities of size 1–68 species (10,000 permutations used for each level of species richness)

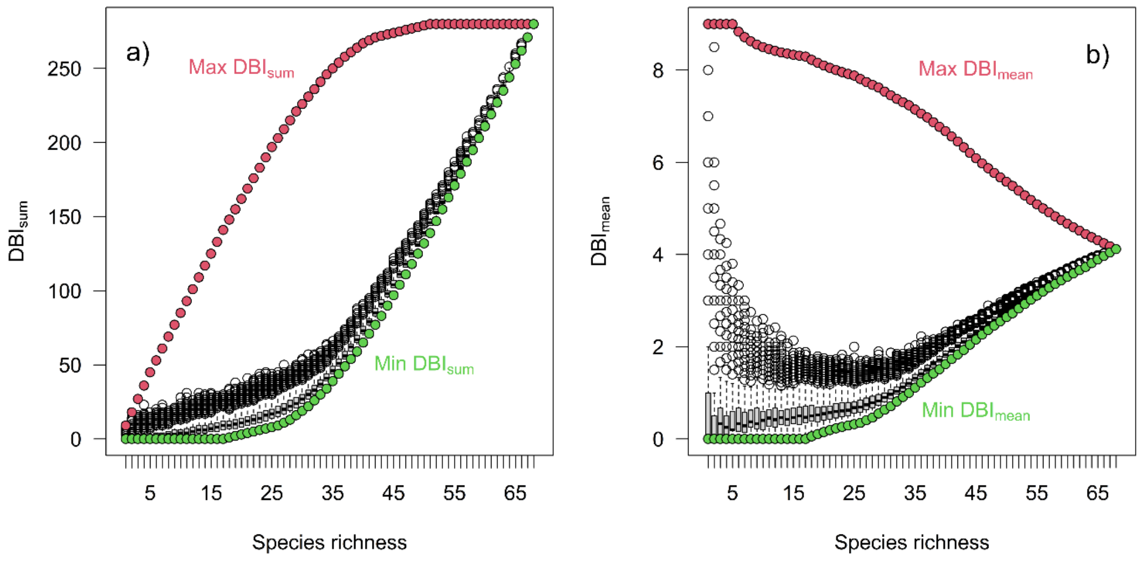

To verify the applicability of probability weight function $P_{DBI} = 2^{-DBI}$ to the South African dataset, we used data from Diedericks et al.[9] (6 randomly selected communities), and Avidon et al. [10] (32 communities). Using the library 'dragDBI'[11] in R 4.2.1[12], we calculated DBI_{sum}, DBI_{mean}, and permutational DBI potential (Fig. S6).

Fig. S6. Odonate communities ranked according to permutational DBI potential calculated by 'dragDBI' package for communities reported by Diedericks et al. (2013) ($pDBI_{min} = 0.622$, $pDBI_{mean} = 0.829$, $pDBI_{max} = 0.984$; (a); and Avidon et al. (2021) ($pDBI_{min} = 0.000$, $pDBI_{mean} = 0.448$, $pDBI_{max} = 0.951$, (b). Relationship between permutational DBI potential and DBI_{mean} (c), and DBI_{sum} (d) for 32 communities reported in Avidon et al. (2021)

[9] Diedericks, G., Simaika, J., & Roux, F. (2013). A Survey of Adult Odonata Along the Crocodile-Inkomati River Main Stem from Source to Ocean. *Report to the Mpumalanga parks and tourism Agency*, 1-56.
[10] Avidon, S. N. (2021). Contextualizing waterscape health in a subtropical rangeland (Master thesis, Stellenbosch University).
[11] https://github.com/VeronikaPrielozna/dragDBI
[12] R Core Team, 2021. R: a language and environment for statistical computing. R Foundation for Statistical Computing, Vienna, Austria. https://www.R-project.org.

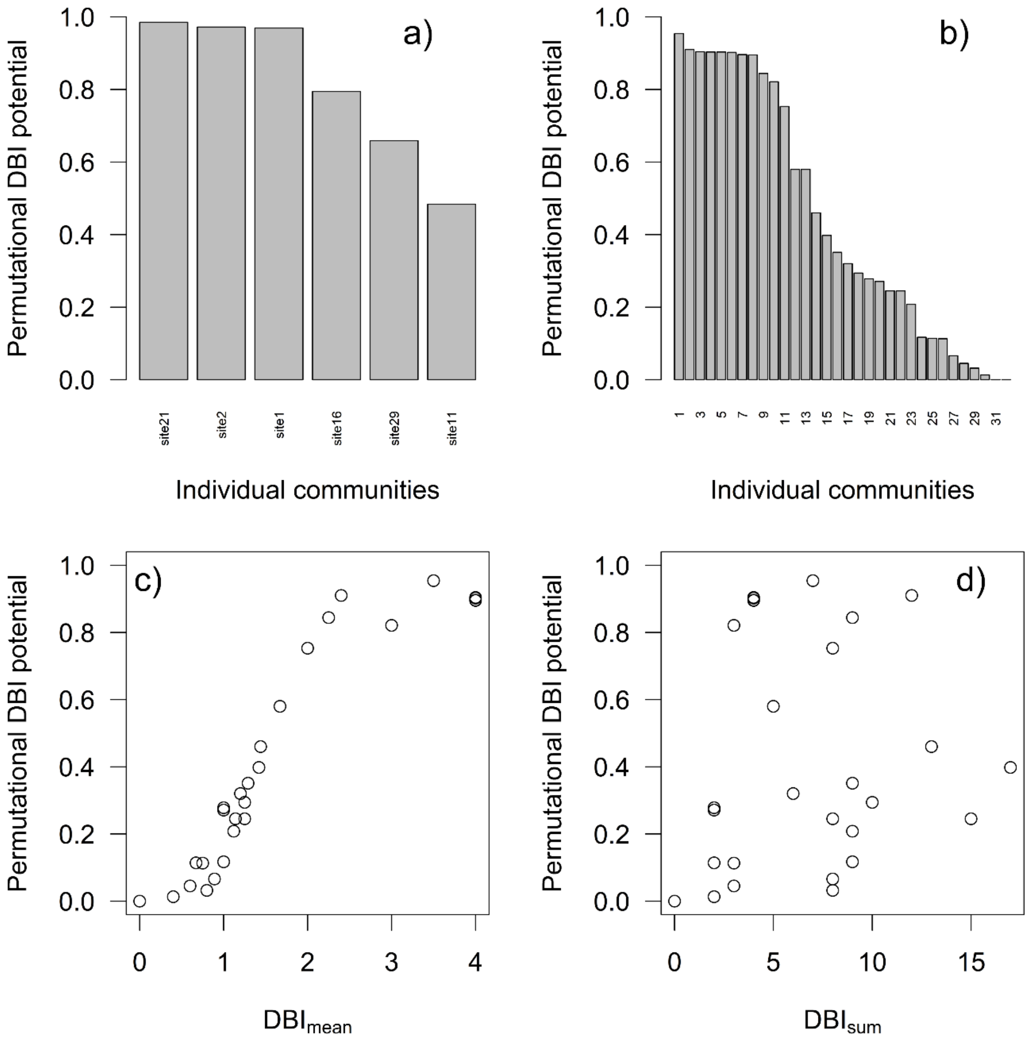

a)
b)
c)
d)
Permutational DBI potential
Individual communities
site21
site2
site1
site16
site29
site11
DBI$_{mean}$
DBI$_{sum}$
